# Informed Consent: An Ethical Obligation or Legal Compulsion?

**DOI:** 10.4103/0974-2077.41159

**Published:** 2008-01

**Authors:** K H Satyanarayana Rao

**Affiliations:** *Central Government Health Scheme, Bangalore, Karnataka, India*

**Keywords:** Informed consent, Ethical obligation, Legal complusion

## Abstract

Informed consent is a vital document while performing all surgical and aesthetic procedures, particularly in the current day practice. Proper documentation and counseling of patients is important in any informed consent.

## INTRODUCTION

Medical practice today is not simple because of various factors impinging on the doctor-patient relationship. Mutual trust forms the foundation for good relationship between doctor and patient. Today, patients tend to be well- or ill-informed about the disease and health. With the hype created in the print and visual media regarding ‘beauty’, ‘shape, size and appearance of body parts’, ‘quality and quantity of hair’, etc., patients tend to come to dermatologists with unreasonable demands and unrealistic expectations. Therefore, providing adequate information and educating the patient about realities and obtaining informed consent before subjecting a patient to any test/procedure/surgery is very essential.

## ETHICAL ANGLE

The concept of consent arises from the ethical principle of patient autonomy[1] and basic human rights.[2] Patient's has all the freedom to decide what should or should not happen to his/her body and to gather information before undergoing a test/procedure/surgery. No one else has the right to coerce the patient to act in a particular way. Even a doctor can only act as a facilitator in patient's decision making.

## LEGAL ANGLE

There is also a legal angle to this concept. No one has the right to even touch, let alone treat another person. Any such act, done without permission, is classified as “battery”[3] - physical assault and is punishable. Hence, obtaining consent is a must for anything other than a routine physical examination.

## CONSENT

In simple terms, it can be defined as an instrument of mutual communication between doctor and patient with an expression of authorization/permission/choice by the latter for the doctor to act in a particular way.

## IMPLIED VS. EXPRESSED CONSENT

The very act of a patient entering a doctor's chamber and expressing his problem is taken as an implied (or implicit) consent for general physical examination and routine investigations. But, intimate examination, especially in a female, invasive tests and risky procedures require specific expressed consent. Expressed (explicit) consent[4,5] can be oral or written. Written consents are preferable in situations involving long-term follow-up, high-risk interventions and cosmetic procedures and surgeries. It is also needed for skin biopsy, psoralen with ultraviolet A (PUVA) therapy, intralesional injection, immunosuppressive therapy, electrocautery etc.[6]

Consent is necessary for photographing a patient for scientific/educational/research purpose or for follow up. Specific consent must be taken if the identity of the patient is likely to be revealed while publishing.[7]

Consent is a must for participation in clinical trials and research projects.[8]

## INFORMED CONSENT

Informed consent must be preceded by disclosure of sufficient information. Consent can be challenged on the ground that adequate information has not been revealed to enable the patient to take a proper and knowledgeable decision. Therefore, accurate, adequate and relevant information must be provided truthfully in a form (using non-scientific terms) and language that the patient can understand. It cannot be a patient's signature on a dotted line obtained routinely by a staff member.

## DISCLOSURE OF INFORMATION

The information disclosed[9] should include:

- The condition/disorder/disease that the patient is having/suffering from
- Necessity for further testing
- Natural course of the condition and possible complications
- Consequences of non-treatment
- Treatment options available
- Potential risks and benefits of treatment options
- Duration and approximate cost of treatment
- Expected outcome
- Follow-up required

Patient should be given opportunity to ask questions and clarify all doubts. There must not be any kind of coercion. Consent must be voluntary and patient should have the freedom to revoke the consent. Consent given under fear of injury/intimidation, misconception or misrepresentation of facts can be held invalid.

## PRE-REQUISITES

Patient should be competent[10] to give consent; must be an adult and of sound mind. In case of children, consent must be obtained from a parent. In case of incapacitated persons, close family members or legal guardians can give consent. Adequate information should be provided to a prudent patient during informed consent.

Prudent patient means a reasonable or average patient. To decide whether adequate information has been given, courts rely on this “Prudent Patient Test”. It is not easy to answer the question, How much information is “adequate”? A netizen may expect and demand detailed information. On the other hand, an illiterate may say that “I do not understand anything, doctor, you decide what is best for me!” If a patient knowingly prefers not to get full information that attitude also needs to be respected as a part of patient's right to autonomy.[11]

Patients' perception of risk of a medical intervention is also highly individualistic, variable and unpredictable. The information provided to a patient should include all material risks. But, the list of risks and side effects cannot be exhaustive to the level of absurdity and impracticality. For example, hardly any patient can go through the product information leaflet included in any drug pack and if some body does, it is unlikely that the drug is consumed. So, what is expected is that the doctor should provide information that a prudent[3] or reasonable patient would expect to make a knowledgeable decision about the course of action to be taken in the presence of alternatives.

## EXCEPTIONS TO DISCLOSURE

### Therapeutic privilege[3,4]

If a doctor is of the opinion that certain information can seriously harm a patient's health - physical, mental or emotional - he has the privilege to withhold such information. But, it should be shared with close relatives. This situation usually does not occur in cutaneous aesthetic surgical procedures.

### Placebo

Use of placebos in certain self-limiting conditions or in patients with high psychological overlay or in those who insist for some form of medication[10] is justified as there are high chances of benefit to the patient with negligible risk. Revealing the truth to the patient takes away the very purpose of administration of placebo.

## BLANKET CONSENT

An all-encompassing consent to the effect “I authorize so and so to carry out any test/procedure/surgery in the course of my treatment” is not valid. It should be specific for a particular event. If, consent is taken for microdermabrasion, it cannot be valid for any other procedure like acid peel. Additional consent will have to be obtained before proceeding with the latter.

If a consent form says that patient has consented to undergo laser resurfacing by Dr. X, the procedure cannot be done by Dr. Y, even if Dr. Y is Dr. X's assistant, unless it is specifically mentioned in the consent that the procedure may be carried out by Dr. X or Dr. Y (or his authorized assistants).

## DOCUMENTATION

It is important to document the process of consent taking. It should be prepared in duplicate and a copy handed over to the patient. It should be dated and signed by the patient or guardian, the doctor and an independent witness. Assisting nurse preferably should not be a witness. Like all other medical records, it should be preserved for at least 3 years.

## INFORMED REFUSAL

Patient has got the right of self-determination. If, a doctor diagnoses varicella in a child, the parent may choose to avail no treatment because of religious belief. Doctor's duty is to explain the possible consequences of non-treatment and benefits of treatment and leave the decision to the parent. Such informed refusals must be documented clearly.[6,10] But, a patient's freedom cannot impinge on the rights of others or cause harm to a third party or community. Therefore, the said parent's freedom of choice cannot extend to sending the child to school, as the infection can spread to other children.

Discharge against medical advice also falls into this category and needs to be properly recorded in the case sheet with signature of the patient/guardian.

In an emergency situation, for example intestinal perforation, a doctor may have to operate even in the absence of consent, to save the life of the patient. It is possible that even with such an intervention, the patient may not survive. Assuming that the doctor is competent and has exercised due care and diligence, doctor cannot be held responsible for patient's death, as he has acted in good faith and in the best interest of the patient. This protection is given under Section 88 of Indian Penal Code.[10]

## CONCLUSION

Obtaining consent is not only an ethical obligation, but also a legal compulsion. The level of disclosure has to be case-specific. There cannot be anything called a standard consent form. No doctor can sit in comfort with the belief that the “consent” can certainly avoid legal liability. This is highlighted by the note of The California Supreme Court:[12]

*“One cannot know with certainty whether a consent is valid until a lawsuit has been filed and resolved.”*

One can only take adequate precaution and act with care and diligence. Maintaining good relationship with patient often works better than the best informed consent!
